# Quorum sensing gene regulation in *Staphylococcus epidermidis* reduces the attraction of *Aedes aegypti* (L.) (Diptera: Culicidae)

**DOI:** 10.3389/fmicb.2023.1208241

**Published:** 2023-06-22

**Authors:** Dongmin Kim, Tawni L. Crippen, Heather R. Jordan, Jeffery K. Tomberlin

**Affiliations:** ^1^Department of Entomology, Texas A&M University, College Station, TX, United States; ^2^Southern Plains Agricultural Research Center, Agricultural Research Service, US Department of Agriculture, College Station, TX, United States; ^3^Department of Biological Sciences, Mississippi State University, Starkville, MS, United States

**Keywords:** microbes, microbial volatile organic compounds, mosquito attraction, interkingdom communication, gene regulation

## Abstract

**Introduction:**

Identifying mechanisms regulating mosquito attraction to hosts is key to suppressing pathogen transmission. Historically, the ecology of the host microbial community and its influence on mosquito attraction, specifically, whether bacterial communication through quorum sensing (QS) modulates VOC production that affects mosquito behavior have not been extensively considered.

**Methods:**

Behavioral choice assays were applied along with volatile collection, followed by GC-MS and RNA transcriptome analyses of bacteria with and without a quorum-sensing inhibitor, furanone C-30.

**Results:**

Utilizing the quorum-sensing inhibitor on a skin-inhabiting bacterium, *Staphylococcus epidermidis*, we disrupted its interkingdom communication with adult *Aedes aegypti* and mitigated their attraction to a blood-meal by 55.1%.

**Discussion:**

One potential mechanism suppressing mosquito attraction could be the reduction (31.6% in our study) of bacterial volatiles and their associated concentrations by shifting *S. epidermidis* metabolic (12 of 29 up regulated genes) and stress (5 of 36 down regulated genes) responses. Manipulating the quorum-sensing pathways could serve as a mechanism to reduce mosquito attraction to a host. Such manipulations could be developed into novel control methods for pathogen-transmitting mosquitoes and other arthropods.

## 1. Introduction

Hematophagous arthropods transmit ~17% of pathogens responsible for human infectious diseases globally (World Health Organization, 2017). Owing to its robust anthropophilic (host preference for humans) and endophilic (strong association with human communities) behaviors, the mosquito *Aedes aegypti* (Linnaeus) (Diptera: Culicidae) is a highly efficient vector of numerous arboviruses, including dengue, yellow fever, Zika, and chikungunya, which have a significant impact on public health (Takken and Knols, 1999). These diseases have spread out of their native country Africa, facilitated by the worldwide spread of *Ae. aegypti* (Powell et al., 2018). Mosquitoes use visual stimuli (Muir et al., 1992), carbon dioxide (Gillies, 1980), heat (Davis and Sokolove, 1975), and volatile organic compounds (VOCs) through [e.g., receptors on the antenna (Davis and Sokolove, 1975), maxillary palps (Lu et al., 2007), and tarsi (Bentley and Day, 1989)] to locate hosts. However, recently, a connection has been made between numerous primary olfactory cues for mosquitoes that have been demonstrated to emanate from microbes associated with a host's skin (Braks et al., 2000; Verhulst et al., 2011; Michalet et al., 2019). Unfortunately, the relevance of VOCs emanating from communication between host commensal microbes to mosquito attraction has not been deciphered yet.

Human skin is an ecosystem consisting of multiple niches occupied by complex microbial (e.g., fungal and bacterial) communities (Grice et al., 2008). Many host-associated factors, such as topographical location, age, immune status, and sex, modulate the composition and function of these communities (Grice and Segre, 2011). This complex set of factors impacts microbial community structure and, in turn, its associated VOC production (Sharon et al., 2010). Many VOCs associated with bacterial metabolic activity were hypothesized to be waste products or evolutionary leftovers (Haslam, 1985), which do not enhance the fitness of the producer (Firn and Jones, 2000). However, these secondary metabolic pathways allow a bacterium broader access to alternative nutrient biosynthesis and present a good strategy for establishment in a new environment or response to stress [i.e., (p) ppGpp, guanosine tetraphosphate alarmone, and guanosine pentaphosphate involved in stringent response], facilitating persistent survival (Breitling et al., 2013). Moreover, specific microbial VOCs provide distinct types of information to eavesdropping flies (Diptera) with differing foraging interests (Liu et al., 2016). In some instances, different VOC profiles guide the decisions of mosquitoes toward different host and oviposition site**s** (Syed, 2015). Thus, this study was initiated to provide a comprehensive understanding of the link between the ecology of host-microbial communities, their communication pathways, and downstream effects on mosquito attraction.

Microbial community VOCs serving as cues regulating mosquito responses to potential hosts are diverse (e.g., alcohols, hydrocarbons, ketones, and short-chain fatty acids) (Schulz and Dickschat, 2007; Korpi et al., 2009; Verhulst et al., 2010, 2011) and vary in community composition across microbial species (Korpi et al., 2009). *Staphylococcus epidermidis* is a predominant commensal bacterium primarily colonizing human epithelia, axillae, head, and nares (Kloos and Musselwhite, 1975; Noble, 2004). It converts odorless sweat secreted from human skin glands (e.g., sebaceous, apocrine, and eccrine) (Kai et al., 2009) into distinct odors (Schulz and Dickschat, 2007; Verhulst et al., 2010, 2011). This species produces a wide spectrum of characteristic VOCs (e.g., short-chain volatile fatty acids) (Schulz and Dickschat, 2007; Verhulst et al., 2010, 2011) through a metabolic emanation that is secreted from human skin glands (e.g., sebaceous, apocrine, and eccrine) (Noble, 2004). Mosquitoes detect these VOCs through their olfactory system to locate, evaluate, and potentially feed on hosts (Schulz and Dickschat, 2007; Verhulst et al., 2010, 2011). Such an ability allows mosquitoes to more effectively forage and improve their fitness by distinguishing between suitable and unsuitable hosts and thus securing a fit forage advantage (e.g., voltinism) (Blackmore and Lord, 2000). Such microbial VOCs provide information to insects foraging for resources (Liu et al., 2016), but the mechanistic effects of specific VOCs on mosquito responses have not been fully delineated.

The team hypothesized that microbial VOC production was tightly linked with bacterial quorum sensing (QS), which coordinates community responses by regulating cellular phenotypical and physiological characteristics [e.g., agr system (Peng et al., 1988) or LuxS (Li et al., 2008) in staphylococci and for system (Mylonakis et al., 2002) in enterococci]. Bacterial QS elicits either an inductive or inhibitory effect for regulating biological activities and ecological fitness in conspecific and heterospecific environments (Rasmussen and Givskov, 2006; He et al., 2012). These responses are related to a host of functions, such as symbiosis (Lupp et al., 2003), virulence (Vuong et al., 2000), conjugation (Dunny et al., 1978), antibiotic production (Bainton et al., 1992), and biofilm formation (Kong et al., 2006). Thus, furanone (Kuehl et al., 2009; He et al., 2012) as a quorum-sensing inhibitor (QSI) that can attenuate QS-controlled behaviors without selective pressure for resistance has been proposed among other bacterial species (e.g., *S. epidermidis*) as a potential strategy to mitigate pathogenicity (Hentzer et al., 2003) and biofilm formation (He et al., 2012).

Interkingdom interactions, such as those between kingdoms Animalia and prokaryotes mediated by QS compounds, have been identified for several systems (Mathesius et al., 2003; Ma et al., 2012; Tomberlin et al., 2012; Zhang et al., 2015), and molecules of the QS machinery have been shown to modulate host immune effects (Chhabra et al., 2003; Ritchie et al., 2003) and mimic virulence regulatory factors (Hartmann et al., 2014). Some QS molecules (e.g., indole) provide distinct types of information to augment multicellular organismal behaviors and responses (e.g., vector host preference and oviposition site selection) (Tomberlin et al., 2012; Zhang et al., 2015; Liu et al., 2016; Mosquera et al., 2023). Our previous research has demonstrated differential detection and response by mosquitoes to wildtype *S. epidermidis* and QS-inhibited (agr-) *S. epidermidis* mutant (Zhang et al., 2015) and blow fly (Diptera: Calliphoridae) attraction to wildtype *Proteus mirabilis*, a commensal mutant, and its rfaL QS-mutant (Ma et al., 2012). These studies allude to a function of volatile QS compounds as active participants, providing eukaryotic organisms [i.e., plants, (in)vertebrates, and arthropods] with the capability of interacting with their landscapes to manipulate behaviors for biological fitness in stochastic ecosystems. However, the mechanisms regulating bacterial engagements with eukaryotes within an environment, such as the mosquitoes' genomic responses to QSIs and the resulting downstream impacts on their behavior, are not defined. Thus, we measured QS pathways within the human commensal bacterium, *S. epidermidis*, with and without a functional inhibitor, by mRNA expression and VOC production. We also linked these physiological responses of the bacteria with the host-seeking behavior of mosquitoes. Such information provides evidence of mosquitoes assessing vertebrate hosts based on commensal microbial communication pathways, which could enhance the selection of suitable hosts and increase the probability of pathogen transmission, thus demonstrating the direct interkingdom connection between bacterial QS regulation and arthropod behavior.

## 2. Materials and methods

### 2.1. Mosquito colony

*Aedes aegypti aegypti* (Liverpool strain) were maintained in a colony held in an environmental chamber (25.0 ± 0.5°C, 65.0 ± 5.0% RH, and a photoperiod (L:D) of 12:12 h) at the Forensic Laboratory for Investigative Entomological Sciences (F.L.I.E.S. Facility) at Texas A&M University, College Station, Texas, USA. Mosquito larvae (~l,000) were reared in enamel pans (25 × 35 × 5 cm) containing 1.5 L of reverse osmosis (RO) water. Larvae were fed a diet of fish food, TetraMin (Tetra, Virginia, USA), on a standardized mosquito-rearing schedule (Gerberg et al., 1994). Pupae were collected daily and placed in a 50-ml cup of RO water at a density of 100 larvae/cup. Containers were divided into groups of three and placed into 30.5 × 30.5 × 30.5 cm aluminum screened wire mesh cages (BioQuip Products Inc., California, USA) for adult eclosion. Emergent adults were provided *ad libitum* with a 10% sucrose solution placed on absorbent cotton rolled in cotton-muslin gauze cloth and inserted in a 50-ml glass bottle placed inside each adult cage. Blood-feeding of 3–5-day-old (post-emergence) female mosquitoes was performed using a 1 ml aliquot of defibrinated rabbit blood (HemoStat Laboratories, California, USA) in an artificial membrane. At 48 h after blood feeding, a 2 × 5 cm filter paper placed in a 50-ml black cup containing 30 ml of RO water was provided as an oviposition site in each cage. Females were allowed to deposit eggs in the container for 3 days. Filter papers containing eggs were removed from the container and placed on a shelf in the incubator room, allowed to air dry, and then stored at room temperature until use.

### 2.2. Bacteria and QSI preparations for mosquito behavior assay

*Staphylococcus epidermidis* (1457) strain (Kies et al., 2003) was grown on mannitol salt agar (MSA; Neogen Corp., Michigan, USA) at 37°C for 48 h, isolated onto a blood agar plate, and then incubated overnight at 37°C. For use in the mosquito behavior experiment, an inoculum of 10^8^ cfu (colony-forming units)/ml (2.1 ± 0.6 × 10^8^ cfu/ml) in phosphate-buffered saline (PBS) was used. Preliminary experiments showed no mosquito interactions induced with the PBS diluent used for *S. epidermidis* stock (Supplementary Figure 1). For QSI, 10 mg (0.01 g) of brominated furanone C-30 ((Z-)-4-Bromo-5-(bromomethylene)−2(5H)-furanone, C5H2Br2O2; Sigma-Aldrich Corp., Missouri, USA) (Chemical FW = 253.88 g/mole) was dissolved in 78.77 ml of methanol. Preliminary experiments showed no mosquito interactions induced by the methanol diluent used for the 500 μM furanone C-30 stock solution (Supplementary Figure 2).

### 2.3. Experiment design for mosquito behavior assay

The experiments were a modification of previously described methods (Zhang et al., 2015). At 2 h prior to each trial, 50 mated female mosquitoes (3–5 d-old post-emergence) that had never been offered a blood meal were collected using a battery-powered aspirator (Hausherrs Machine Works Co., New Jersey, USA). Mosquitoes were released into a clear Plexiglas^®^ cage (82 × 52 × 45 cm) with a wire mesh top and allowed to acclimate at a temperature of 25.0 ± 0.5°C and relative humidity of 65.0 ± 5.0%. Experiments were performed 30 min after sunrise (chamber at 12:12 L:D), which corresponded to the normal biting activity of *Ae*. *aegypti* (Yasuno and Tonn, 1970).

Blood feeders were individually constructed from a 25-ml sterile tissue culture flask (Corning Inc., New York, USA) tightly wrapped with parafilm and secured with cellophane tape. A 1-ml aliquot of defibrinated rabbit blood (HemoStat Laboratories, California, USA) was injected into the space between the culture flask and parafilm. A 5.0 × 5.0 cm piece of sterilized 100% cotton gauze (Dynarex Co., South Carolina, USA) was placed to absorb the inoculum over the parafilm and secured with two autoclaved rubber bands.

Dual choice assays were performed with two blood feeders placed at equal distances horizontally and vertically apart with the cotton gauze inoculated with 1 ml of (1) H_2_0 (reverse osmosis water) or PBS (phosphate-buffered saline), (2) with or without MeOH (methanol), (3) with or without 50 μM/ml QSI, and (4) with or without 10^7^ cfu/ml *S. epidermidis* and 50 μM/ml QSI side-by-side but separated by a parafilm dam to prevent mixing or physical contact (NSE-QSI). Each treatment solution was applied directly to the gauze 15 min before the experiment. For each trial, three or four replicates were performed in succession by rotating each of the two treatments to prevent positional bias.

For quadruple choice assays, four blood feeders were placed at equal distances horizontally and vertically (24 cm) apart in a square pattern, with the gauze side down on the wire mesh top while connected to a water bath (Thermo Fisher Scientific, Connecticut, USA), and maintained at 37°C (Zhang et al., 2015). Cotton gauze assigned to each replicate on a blood feeder was inoculated with 1 ml of either 10^7^ cfu/ml *S. epidermidis* (SE), 50 μM/ml QSI (QSI), 10^7^ cfu/ml *S. epidermidis* + 50 μM/ml QSI (SE+QSI), or only PBS (CONT) as a control. Each treatment solution (i.e., QSI and bacteria) was mixed in a 1.5-ml microfuge tube 15 min before application to the gauze. The effect of 10^7^ cfu/ml *S. epidermidis* + 50 μM/ml QSI that was neither mixed nor in physical contact (NSE-QSI), QSI alone, or solvent controls (PBS for *S. epidermidis* and methanol for QSI) on mosquito responses was also measured using a dual choice assay (methods provided in Supplementary material). For each trial, four replicates were performed in succession by rotating each of the four treatments to each of the four different corner locations initially assigned by a random number generator and rotated clockwise across trials to prevent positional bias (*n* = 12). The Plexiglas^®^ cages were cleaned with 3% Lysol and 95% ethanol and allowed to dry and air out between trials. During the experiments, mosquito landing and probing activity at each blood feeder was recorded on video, with no one present in the room during the trial, with cameras (2160 p/30 fps, LG, Korea) mounted on the outside of the cage. The number of ladings and probings of duration at each blood feeder (response) longer than 1 s were recorded over a 15-min assay period. A particular mosquito feeding blood on one of the treatments was continually counted at each second, which reduced a frequency variation biased in the total number of mosquitoes. This matrix, modified from the human landing catch (HLC) technique as a gold standard tool for mosquito host-seeking (landing) behavior (Shirai et al., 2002), engaged in sequential behaviors, including piercing and blood feeding on hosts, which provided a direct and sensitive estimate of mosquito behavioral responses and additionally intraspecific interaction over a given period.

### 2.4. Statistical analysis for the mosquito behavior assay

The normality of the data was determined for the number of landings and probings by mosquitoes over a 15-min assay period as a function of treatment using the Shapiro–Wilk test (*P* ≤ 0.05 rejected normal distribution). All statistical procedures were conducted using JMP Statistics, Version 15.0 (SAS Institute Inc., North Carolina, USA). The alpha was set at 0.05 for all statistical tests. The odds ratios of choosing a particular treatment applied in a blood feeder were tested using R version 3.4.3 and used the DescTools package (https://cran.r-project.org/web/packages/DescTools/index.html). Generalized linear mixed models (GLMM) were performed using R version 3.4.3, with response variables being the number of landings and probings by *Ae. aegypti* with Poisson family distribution to the different treatments used as fixed effects, experimental groups (trials) as a random effect, and time as a covariate. We considered a repeated measures design with an autoregressive (1) covariance structure. The models were validated through the exploration of residual errors with graphical tools and overdispersion access (Zuur et al., 2010; Hartig, 2017). Nonparametric tests with either Wilcoxon's test or the Kruskal–Wallis test followed by the Steel-Dwass test were used to measure differences in mosquito behavioral responses, grouped by replicates, among treatments.

### 2.5. Experiment design for microbial VOCs collection assay

Bacterial volatiles were analyzed in triplicate experiments from the following samples in PBS as a diluent: (1) 0.5 ml of 10^7^ cfu/ml *S. epidermidis*; *(*2) each 0.5 ml of 50 μM/ml QSI plus 10^7^ cfu/ml *S. epidermidis*; (3) 0.5 ml of 50 μM/ml QSI; and (4) 0.5 ml of PBS. The protocol was modified by Groenhagen et al. (2013) and designed to improve filtration in the incoming air. Each sample was transferred to a 12-ml amber glass bottle, and VOCs were collected by the closed-loop-stripping-analysis (CLSA) technique at room temperature. Before every headspace sampling from treatment, the apparatus was thoroughly cleaned with dichloromethane (CH_2_Cl_2_) and autoclaved at 121°C for 15 min. Each amble glass bottle was placed in a 7.5 × 11-cm (O.D × H) glass filtering jar (Kimble Chase, New Jersey, USA) with a flat ground glass and sealed with a parafilm. The rubber stopper on the top of the glass filtering jar was equipped with one hole and inserted with a volatile trap packed with ~30.0 mg of Hayesep^®^ Q porous polymer (Volatile Assay Systems, New York, USA), connecting a vacuum pump (Rocker, Scientific Co., Ltd., New Taipei City, Taiwan) with Tygon^®^ tubing (Saint-Gobain S.A., Pennsylvania, USA). The tooled hose was connected with 3 cm of Tygon^®^ tubing piece inserted with a bacterial filter (Midwest Supplies, Minnesota, USA, 0.2 μm pore size) and a 14.6-cm carbon-filtered pipet (Marineland, Ohio, USA) to purify incoming air. Samples from each treatment were obtained by running the apparatus at 1 L min^−1^ for 1 h. Samples were added with an additional 5.0 μl of 80 ng/μl n-octane (Sigma-Aldrich, MO, USA) as an internal standard and stored at −20°C until analysis.

### 2.6. GC-MS analysis

GC-MS analyses were carried out on an Agilent 6890 Gas Chromatograph with an Agilent Technologies 5973N Mass Selective Detector (Agilent Technologies, California, USA) by the Environmental Research Group at Texas A&M University in College Station, Texas. The GC was programmed as follows: 5 min at 50°C, increasing at 5°C/min^−1^ to 320°C, and operated in split/splitless mode: 60 s at 250°C. A carrier gas, helium, was used at 1.2 ml min^−1^. Candidate identification of compounds was made by matching the comparison of mass spectra with the mass spectra fragmentation patterns in the National Institute of Standards and Technology (NIST) 05 mass spectra library for peaks observed in the chromatograms.

### 2.7. Statistical analysis for microbial VOCs

The GC-MS data were processed to estimate the percentage of the area of each compound in every sample across treatments, including control. To determine the difference among volatile profiles, permutational multivariate analysis of variance (PERMANOVA) was tested using the Adonis function in R version 3.4.3, vegan package (http://CRAN.R-project.org/package=vegan). VOC profiles were analyzed using non-metric multidimensional scaling (NMDS) based on the Bray–Curtis distance matrix to minimize the complex data of the area percentages in a two-dimensional space. An indicator species analysis was conducted to identify the compounds as influential species that may or may not be related to each group. The reliability of stress values was set at < 0.2. Compound abundance was also compared using a two-way ANOVA using JMP^®^ statistical software version 13 (SAS Institute Inc., North Carolina, USA) and the Tukey-Kramer Honestly Significant Difference (HSD). Significant levels were set at a *P*-value of ≤ 0.05.

### 2.8. Experiment design for transcriptome analysis assay

*Staphylococcus epidermidis* was grown as described above. Three replicates of 3 mL frozen (−80°C) 10^7^ cfu/ml bacterial suspensions in 30% glycerol in tryptic soy broth per treatment (*S. epidermidis* exposed to furanone: SE + fur1, SE + fur2, and SE + fur3) or controls in diluent only (*S. epidermidis* wildtype alone: SEwt1, SEwt2, and SEwt3) were centrifuged at 7,000 × g at 4°C for 5 min. The culture supernatant was removed, and total RNA was isolated from the bacterial pellet using Trizol^®^ (Thermo Fisher Scientific, Massachusetts, USA), following the manufacturer's instructions. Following this, RNA was treated with Turbo DNAse (Thermo Fisher Scientific, Massachusetts, USA) according to the manufacturer's instructions to remove trace DNA. RNA quality was analyzed by agarose gel electrophoresis, and RNA concentrations were determined using Qubit 2.0 (Thermo Fisher Scientific, Massachusetts, USA). All samples were stored at −80°C until further processing for library preparation. Total RNA libraries were created using the NEBNext^®^ Ultra™ RNA Library Prep Kit and NEBNext^®^ Multiplex Oligos (Dual Index Primers) (New England Biolabs, Massachusetts, USA) for Illumina^®^ (Illumina, Inc., California, USA) and associated protocols. High-throughput RNA sequencing was performed by St. Jude Children's Research Hospital on an Illumina HiSeq2000 with 2 × 100 bp PE (paired-end) read lengths.

### 2.9. Statistical analysis for the transcriptome

Sequences were initially trimmed with the sequencing facility using TrimGlare v0.4.2 (Krueger, 2015), but a more stringent quality trimming was also performed using default parameters within the Qiagen CLC Workbench 12.0 (https://www.qiagenbioinformatics.com/) following the QC analysis of sequence reads. The results from high-quality reads were aligned to the *S. epidermidis* (SE) 1457 genome (downloaded from the NCBI database using accession numbers CP020462 and CP020463 corresponding to the *S. epidermidis* genome and plasmid, respectively).

RNASeq data were mapped with the following parameters: (a) maximum number of allowed mismatches was set at 2, with insertions and deletions set at 3; (b) length and similarity fractions were set to 0.8, with autodetection for both strands; and (c) a minimum number of hits per read was set to 10. Gene expression values were reported as reads per kilobase of transcript per million (RPKM) mapped reads. Treatment reads with an absolute fold change of 1.5 and a *p*-value of ≤ 0.5 were considered significant. Following this, transcripts were further annotated into pathways by linking protein ID with potential conserved domains and protein classifications archived within the Conserved Domain Database (Marchler-Bauer et al., 2017) and by using the KEGG and STRING databases (Jensen et al., 2008; Kanehisa et al., 2016). A heat map of expression values was created in the CLC workbench, measuring Euclidean distance with average cluster linkage. Gene expression was filtered with *t*-test statistics using Bonferonni corrected *p*-values with the minimum absolute fold change set at 1.5 and the *P*-value set at ≤ 0.05 for significant expression between *S. epidermidis* wild-type and QSI treatments.

## 3. Results

### 3.1. Mosquito behavior

Preliminary dual choice assays determined that the mosquito responses to blood feeders showed no significant difference (*P* > 0.7575) in responses to blood feeders with H_2_O or PBS over the 15-min period (Supplementary Figure 1), indicating no attraction to the diluent PBS. There was no significant difference between mosquito responses to the blood feeders treated with or without MeOH (*P* > 0.3856), indicating no bias when using MeOH as a QSI solvent (Supplementary Figure 2). While initial dual choice results confirmed no bias in mosquito responses to solvent controls MeOH or PBS, a response was elicited in blood-feeders with QSI alone or NSE–QSI (not mixed and not in physical contact) (Supplementary Figures 3, 4).

In a comparison between blood feeders with or without QSI, a significant difference (*P* ≤ 0.0001) was found in mosquito responses over time, as the total number of responses to blood feeders treated with QSI was 51.7% higher than those to a blood feeder alone (Supplementary Figure 3) indicating some attraction of mosquitoes to QSI over a blood feeder alone. Bacterial VOCs mix through indirect gas diffusion by co-cultivation, without physical contact, of the bacteria that produce them. This can result in new product formation or differential functionality, such as upregulation or downregulation of antimicrobial activity (Singh, 2011; Tyc et al., 2015; Lammers et al., 2022). Therefore, mosquito interactions with blood feeders treated with or without SE and QSI on the same feeder but not mixed (NSE-QSI) were evaluated. The mosquito responses to these NSE-QSI feeders were significantly greater over the 15-min period (*P* ≤ 0.0443) than those to blood feeders alone. The total number of attraction responses [1,449 from the three experiments of triplicate trials (*n* = 9)] to blood-feeders treated with or without NSE-QSI were 800 (55.2%) and 649 (44.8%), respectively (Supplementary Figure 4). Blood-feeders with NSE-QSI elicited a greater response (23.2% in the total number of responses) at all time points compared to the blood-feeder solvent controls, indicating some attraction of mosquitoes to NSE-QSI. This could be owing to the attraction of the mosquitoes to the SE, as observed in the previous pairwise experiment, the QSI, or to both. The mechanism of action of the halogenated furanone C-30 is due to its similar structure to that of quorum sensing autoinducers, allowing it to bind to the QS response regulator while failing to activate it, resulting in inhibition of the QS system (Markus et al., 2021). It is unclear whether this would occur through gas diffusion, but the results of this experiment do not support that conclusion under these specific experimental parameters. The pairwise assays offered limited choices for the mosquitoes that may have skewed the attraction when no other options were presented but allowed us to exclude concerns of possible repellency as these results showed no evidence for QSI-induced mosquito repellency. However, the results required further delineation of mosquito attraction to SE vs. QSI-treated SE; therefore, quadruple choice assays were conducted to parse out the stronger attraction behavior when a wider variety of choices were offered simultaneously.

**Figure 1:**
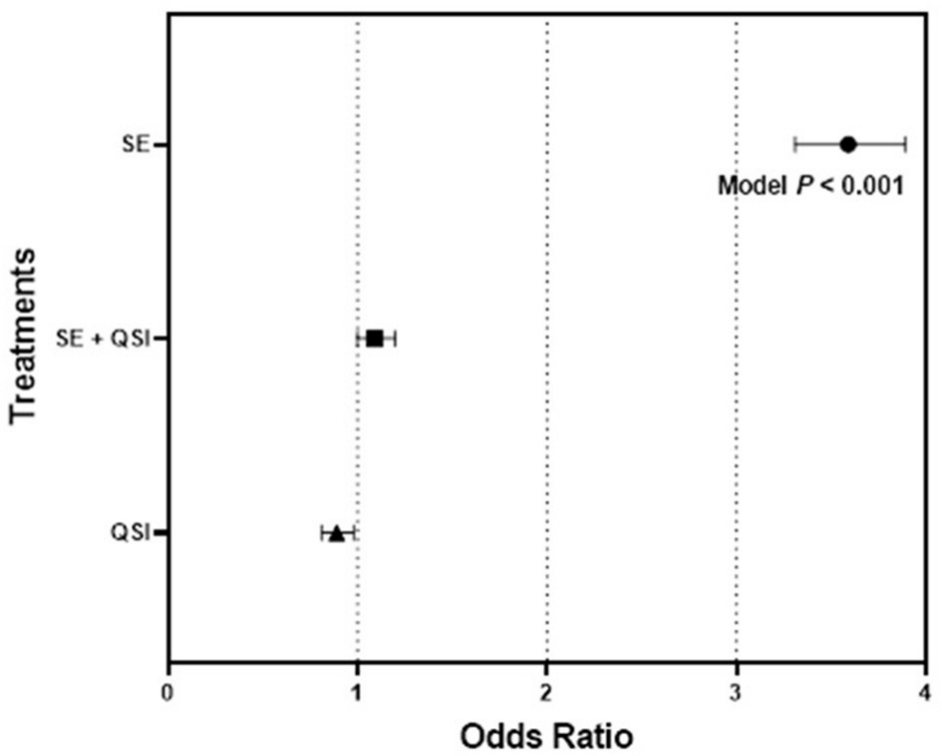
Quadruple choice assay odds ratio. The odds ratios with 95% confidence intervals of 50 mated 3–5-day-old (post-emergence) female *Ae. aegypti* mosquito attraction responses to blood feeders treated with (1) *S. epidermidis* 1457 (SE), (2) SE + QSI (quorum sensing inhibitor furanone C-30), and (3) QSI vs. PBS control placed at equal distance horizontally and vertically (24 cm) apart on the top of an 82 cm (L) × 45 cm (W) × 52 cm (H) Plexiglas^®^ cage during triplicate trials of 15 min conducted at 25.0 ± 0.5°C with 65 ± 5.0% RH, performed 30 min after sunrise (chamber at 12:12 L:D), which corresponded to the normal activity of *Ae. aegypti*. Three trials of quadruplicate replicates were completed (*n* = 12).

**Figure 2:**
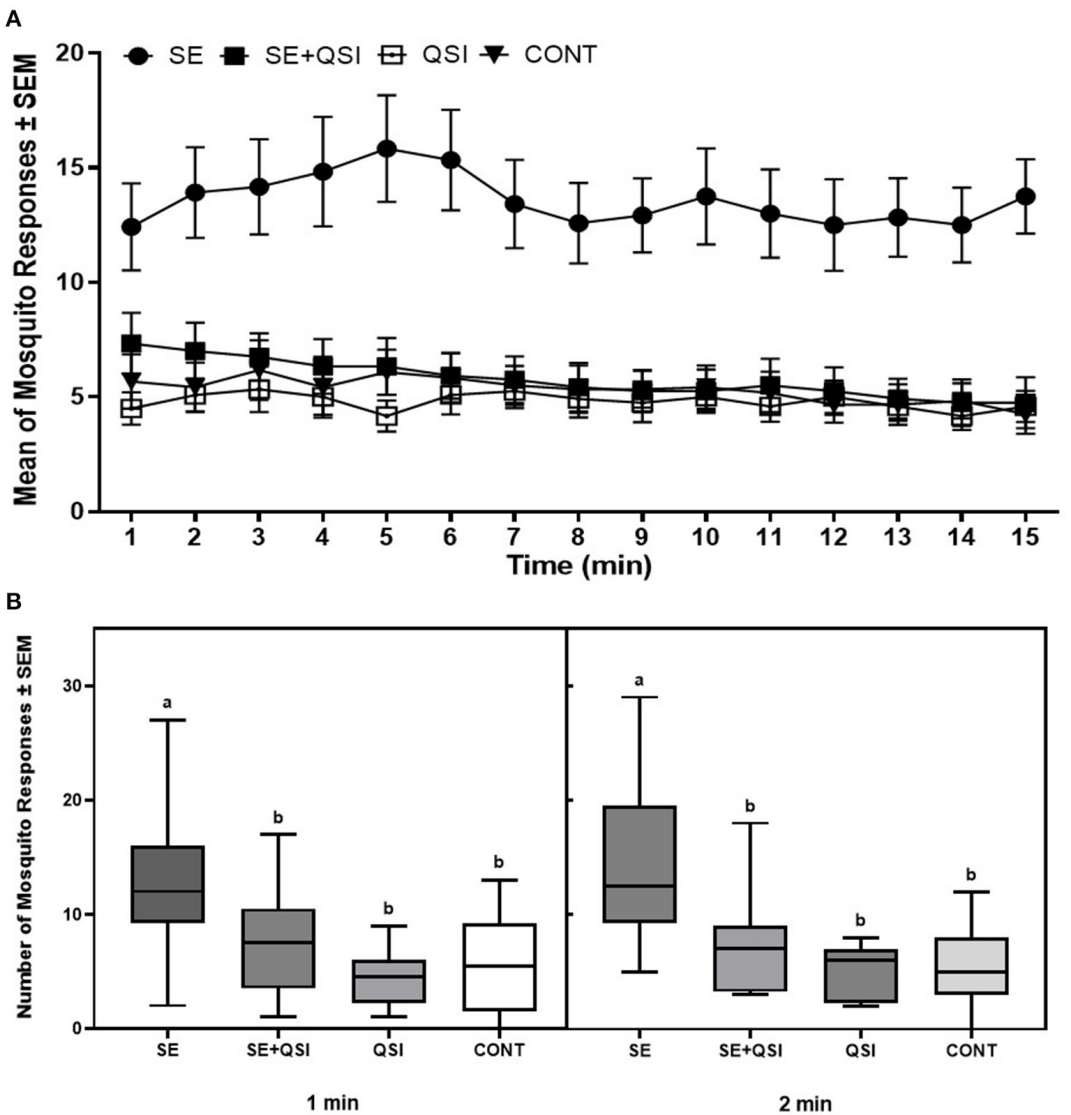
Quadruple choice assay over time. **(A)** The mean of the total number of 50 mated 3–5-day-old (post-emergence) female *Ae. aegypti* mosquito responses per minute ± SEM to blood-feeders treated with *S. epidermidis* 1457 (SE), SE + QSI (quorum sensing inhibitor furanone C-30), QSI, and PBS (CONT) placed at equal distances horizontally and vertically (24 cm) apart on the top of an 82 cm (L) × 45 cm (W) × 52 cm (H) Plexiglas^®^ cage. During each experiment, triplicate trials of 15 min at 25 ± 0.5°C with 65 ± 5.0% RH were performed 30 min after sunrise (chamber at 12:12 L:D), which corresponded to the normal activity of *Ae. aegypti*. Three trials of quadruplicate replicates were completed (*n* = 12). **(B)** Box plots of *Ae. aegypti* mosquito responses during the initial 1 and 2 min of the trials (black line, median; bounds of boxes, first and third quartiles; bars, range). a, b Samples marked with the same letter are not significantly different (*P* ≤ 0.05).

**Figure 3:**
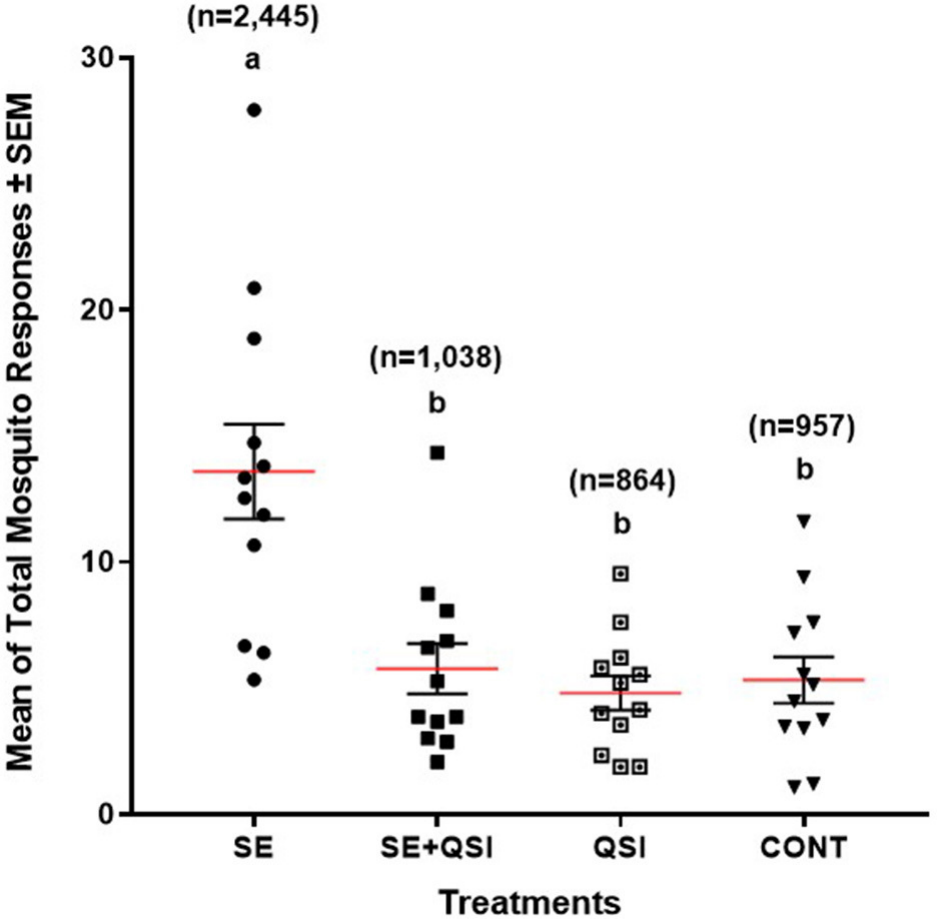
Quadruple choice assay total response. The mean of the total number of 50 mated 3–5-day-old (post-emergence) female *Ae. aegypti* mosquito responses ± SEM to blood-feeders treated with *S. epidermidis* 1457 (SE); *n* = 2,445, SE + QSI (quorum sensing inhibitor furanone C-30); *n* = 1,038, QSI; *n* = 864, and PBS (CONT); *n* = 957 placed at equal distance horizontally and vertically (24 cm) apart on an 82 cm (L) × 45 cm (W) × 52 cm (H) Plexiglas^®^ cage. During each experiment, triplicate trials of 15 min at 25 ± 0.5°C with 65 ± 5.0% RH were performed 30 min after sunrise (chamber at 12:12 L:D), which corresponded to the normal biting activity of *Ae. aegypti*. Three trials of quadruplicate replicates were completed (*n* = 12). Each dot represents an individual mean for a single replication (*n* = 12). The solid red lines and vertical lines indicated a group of means and a standard error, respectively. The same letter is not significantly different (*P* ≤ 0.05).

By determining the odds ratio of mosquito attraction to blood feeders treated with fully functional *S. epidermidis* and *S. epidermidis* with QS inhibited by brominated furanone C-30, we aimed to capture behavioral alterations that are directly linked to bacterial QS functionality. The analysis indicated the mosquito response to the blood-feeders treated with *S. epidermidis* (3.58) was significantly greater than the treatment of *S. epidermidis* + QSI (quorum sensing inhibitor) (1.24) or QSI alone (0.89) relative to the PBS control (Figure 1). While treatments (*S. epidermidis, S. epidermidis* + QSI, QSI, and PBS) (*P* ≤ 0.0001) and time (*P* ≤ 0.0071) significantly impacted mosquito response, no significant trial and time interactions (*P* ≤ 0.3398) were measured. In general, mosquitoes spent 2.36-, 2.83-, and 2.55-fold more time on the blood feeders treated with *S. epidermidis* than with *S. epidermidis* + QSI, QSI, or PBS, respectively, at every time point over the 15-min experimental period (Figure 2). Overall, *S. epidermidis* alone accounted for 46.1% of the total responses, and the inhibition of QS with the addition of QSI to *S. epidermidis* accounted for 19.6% of the recorded responses or 55.1% fewer responses than *S. epidermidis* alone (Figure 3). The QSI compound alone was equally as attractive as *S. epidermidis* + QSI, as well as the PBS control, indicating that the QSI was not repellent. No significant difference was measured between *S. epidermidis* + QSI, QSI, and PBS over time, although mosquito responses to *S. epidermidis* + QSI accounted for 20.1%, which was 8.5% greater than QSI or PBS individually. The results of this assay directly linked QS functionality to a change in mosquito behavior, thereby providing support for the hypothesis that prokaryote QS mechanisms are monitored and interpreted by eukaryotes.

### 3.2. Microbial VOC composition

Mosquitoes use olfactory cues in part to interpret their surroundings (Davis and Sokolove, 1975; Gillies, 1980; Bentley and Day, 1989; Lu et al., 2007), and bacteria produce a multitude of volatiles (Schulz and Dickschat, 2007). The previous assay determined that the application of a QSI to *S. epidermidis* significantly reduced mosquito attraction to a blood meal. By determining the production of VOCs from fully functional *S. epidermidis* and *S. epidermidis* inhibited by QSI, alterations in volatile production directly linked to bacterial QS functionality were captured. A total of 26 compounds were identified by comparing experimental mass spectra with the NIST14 Mass Spectral Library from the headspace volatiles among samples in PBS as a diluent: (1) 10^7^ cfu/ml *S. epidermidis*; (2) 50 μM/ml QSI plus 10^7^ cfu/ml *S. epidermidis*; (3) 50 μM/ml QSI; and (4) PBS (Table 1). Excluding octane, which was added as an internal standard, 19 compounds were detected from *S. epidermidis*, of which 13 were from *S. epidermidis* inhibited by QSI. *Staphylococcus epidermidis* had a mean of 16.00 ± 1.53 compounds compared to 12.00 ± 1.00 with QSI application. The mean number of compounds detected from each of the QSI and control was 13.33 ± 0.33 and 13.67 ± 0.88, respectively. Based on the relative frequency and abundance across treatments and excluding octane, nine VOCs were shared by all treatments: furfural; benzene, 1,3-dimethyl; benzaldehyde; phenol; nonanal; benzothiazole; 2,5 cyclohexadiene; butylated hydroxytoluene; and diethyl phthalate. A total of 19 total compounds were identified from *S. epidermidis*, 10 of which were common to the PBS control. Major changes (>10% difference) in the relative abundance of VOC from SE once exposed to QSI were measured (Table 1), demonstrating a decrease in the proportion of furfural (−45%), benzaldehyde (−39%), phenol (−54%), and butylated hydroxytoluene (−80%) and an increase in the proportion of 1-heptene,4-methyl (24%), and 2,5 cyclohexadiene (40%). Of the compounds not common to PBS, 8 were completely inhibited by the addition of QSI to *S. epidermidis*: 1-heptene,4-methyl; 4,7-methano-1H-indene; pregnane-3d; morphine; octacosane; heneicosane; heptadecane 9-octyl; tetracosane; and lanosta.

**Table 1:** Comparison of relative abundance of compounds produced.

		**Relative abundance**^**a**^ **(mean** ±**SEM) of VOC**								
**#**	**Compound**	**SE** + **QSI**^b^	**SE**	**% diff**	**QSI**	**% diff**	**CONT**	**% diff**	**Retention time (min)**	**Class**
1	Octane^c^	1.0000 ± 0.0000	1.0000 ± 0.0000	0	1.0000 ± 0.0000	0	1.0000 ± 0.0000	0	6.18	Alkanes
2	Furfural	0.0881 ± 0.0286	0.1592 ± 0.0578	45	0.0964 ± 0.0590	9	0.0329 ± 0.0114	−168	7.38	Furan Alcohols Furans Ethers
3	Benzene, 1,3-dimethyl	0.2081 ± 0.0768	0.2187 ± 0.0206	5	0.1338 ± 0.0739	−56	0.1246 ± 0.0838	−67	8.87	
4	1-Heptene,4-methyl	0.0289 ± 0.0254	0.0234 ± 0.0405	−24	0.0000 ± 0.0000	0	0.0000 ± 0.0000	0	11.77	
5	4,7-Methano-1H-indene	0.0000 ± 0.0000	0.0005 ± 0.0003	+	0.0000 ± 0.0000	0	0.0000 ± 0.0000	0	11.86	
6	Benzaldehyde	0.0436 ± 0.0379	0.0710 ± 0.0088	39	0.0561 ± 0.0182	22	0.0261 ± 0.0226	67	12.39	Benzenoids Alcohols Ketones Aldehydes
7	Phenol	0.1080 ± 0.0888	0.2334 ± 0.2026	54	0.2142 ± 0.3631	50	0.0365 ± 0.0584	−196	12.94	Alcohols Benzenoids
8	Benzene,1,3-dichloro	0.0113 ± 0.0113	0.0000 ± 0.0000	–	0.0282 ± 0.0235	60	0.0029 ± 0.0038	−290	14.34	
9	4Cyanocyclohexene	0.0220 ± 0.0176	0.0000 ± 0.0000	–	0.0052 ± 0.0041	−323	0.0005 ± 0.0004	−4,300	14.45	
10	Acetophenone	0.0048 ± 0.0083	0.0000 ± 0.0000	–	0.0055 ± 0.0096	13	0.0000 ± 0.0000	0	16.08	Benzenoids Ketones
11	Nonanal	0.1095 ± 0.0414	0.1122 ± 0.1042	2	0.1075 ± 0.0207	−2	0.0758 ± 0.0545	−44	17.33	Aldehydes
12	Benzothiazole	4.1144 ± 1.9778	3.8326 ± 0.6723	−7	3.5328 ± 0.9384	−16	2.5727 ± 0.4857	−60	21.26	Benzenoids Thiazole Sulfur compound
13	Pentadecane	0.0000 ± 0.0000	0.0000 ± 0.0000	0	0.0001 ± 0.0002	+	0.0008 ± 0.0014	+	25.88	Acids Carboxylic Acids
14	Hexacosane	0.0000 ± 0.0000	0.0000 ± 0.0000	0	0.0443 ± 0.0383	+	0.0000 ± 0.0000	0	25.99	Alkanes
15	2,5 Cyclohexadiene	0.4798 ± 0.3139	0.3429 ± 0.0394	−40	0.4191 ± 0.2007	−14	0.2206 ± 0.0944	−117	27.69	
16	Butylated Hydroxytoluene	0.6653 ± 0.6000	3.3124 ± 3.4150	80	2.7193 ± 2.1180	76	1.8750 ± 2.1527	65	28.69	Benzenoids Alcohols
17	Diethyl Phthalate	0.1090 ± 0.0998	0.1176 ± 0.1023	7	0.0516 ± 0.0446	−111	0.0693 ± 0.0277	−57	30.71	Alcohols
18	Pregnane-3^d^	0.0000 ± 0.0000	0.8623 ± 0.8194	+	0.0000 ± 0.0000	0	0.0000 ± 0.0000	0	37.89	
19	Morphine	0.0000 ± 0.0000	0.7712 ± 0.7007	+	0.0000 ± 0.0000	0	0.0000 ± 0.0000	0	40.65	
20	Octacosane	0.0000 ± 0.0000	0.3665 ± 0.6260	+	0.0001 ± 0.0001	+	0.0000 ± 0.0000	0	42.16	Alkanes
21	Heneicosane	0.0000 ± 0.0000	0.6563 ± 1.1209	+	0.0000 ± 0.0000	0	0.0000 ± 0.0000	0	45.64	Alkanes
22	Heptadecane 9-octyl	0.0000 ± 0.0000	1.5075 ± 1.4871	+	0.0000 ± 0.0000	0	0.0000 ± 0.0000	0	48.25	
23	Tetracosane	0.0000 ± 0.0000	1.0052 ± 1.6381	+	0.0000 ± 0.0000	0	0.0000 ± 0.0000	0	49.79	Alkanes
24	Lanosta^e^	0.0000 ± 0.0000	0.6936 ± 1.1076	+	0.0000 ± 0.0000	0	0.0000 ± 0.0000	0	49.85	
25	Hexadecanoic acid	0.0000 ± 0.0000	0.0000 ± 0.0000	0	0.0000 ± 0.0000	0	0.5189 ± 0.4699	+	50.79	
26	Octadecanoic acid.2	0.0000 ± 0.0000	0.8952 ± 0.7835	+	0.0000 ± 0.0000	0	2.2514 ± 1.9807	+	53.53	

^a^The most abundant octane as an internal standard is assigned 1 and the others assigned a fractional percent of that value.

^b^QSI: quorum sensing inhibitor, (Z-)-4-Bromo-5-(bromomethylene) -2(5H)-furanone.

^c^Compound present.

^d^Pregnane-3: pregnane-3 20-dione.

^e^Lanosta: 20. Xi.-Lanosta-7, 9(11)-diene-3.beta., 18, 20-triol.

Relative abundance of compounds ± SEM identified using GC-MS emitted from *Staphylococcus epidermidis* 1457 (SE) or SE with a quorum sensing inhibitor (SE+QSI), QSI, and control phosphate buffered saline (CONT) from triplicate trials of quadruplicate replicates (*n* = 12) at 25 ± 0.5°C with 65 ± 5.0% RH. The percent change in the VOC detected in comparison to the SE+QSI sample is presented (% diff). Numerical values of up and down regulation were calculated and no change in VOC value detected (0); the VOC was present in this treatment, but not present in SE + QSI (+); and the VOC was not present in this treatment but was present in SE + QSI (–) are also presented.

The eight volatiles from *S. epidermidis* inhibited by treatment with QSI have a variety of functions. For example, 4,7-methano-1H-indene (4,7-methanoindene) has been used in plasticizers, as an ingredient in synthetic waxes and resins and perfume materials or as an intermediate for drugs and insecticides. Novel 4,7-methanoindene derivatives substituted with esters or acids have been patented as useful perfume ingredients (US3557188A United States) and described as having a pleasant odor (Dunkel, 1971). Pregnane-3 (pregnane-3 20-dione) is a steroid hydrocarbon in a form that can be modified to produce several GABA-modulating hormones, including progesterone and other steroid forms capable of urinary excretion. Morphine, an opioid, is known for its analgesic properties. Endogenous opioids can, through the involvement of quorum sensing circuitry, enhance virulence pathways in bacterial pathogens (Zaborina et al., 2007). Heneicosane is a bioactive compound that has been isolated from plants (e.g., *Periploca laevigata*. Labill., *Plumbago zeylanica*, L.) and exhibits antimicrobial capabilities against fungus and gram-positive and negative pathogenic bacteria (Vanitha et al., 2020). It is also an insect oviposition pheromone that can attract or repel gravid female mosquitoes dependent on its concentration (Seenivasagan et al., 2009). Octacosane, heptadecane 9-octyl (9-octylheptadecane), and tetracosane are all alkane hydrocarbons found in plants. Octacosane is a constituent of wood oils that has antimicrobial and cytotoxic activities (Martins et al., 2015). Heptadecane 9-octyl has the potential to be used as an antifungal agent (Abubacker and Devi, 2014). Tetracosane is also a component of the sex pheromone bouquet of the female mining bee [*Andrena nigroaenea* (Kirby) (Francke and Schulz, 2010)]. Lanostane [20.xi.-lanosta-7, 9(11)-diene-3.beta.,18, 20-triol], a triterpenoid, is a polycyclic hydrocarbon. Triterpenoids are common in fungi, marine organisms, and higher plants and participate in the environmental defense mechanisms of the organism. Lanostane specifically has anti-inflammatory and anti-peroxidative properties (Ríos et al., 2000). Therefore, many of these compounds have already been described by their ecological olfactory influences, which, when disrupted by QSI treatment, would presumably affect those appraising their environment through these volatile occurrences.

The relative abundance and quantity range percentages for each treatment are summarized in Table 2. VOCs have been organized by retention times. The differential VOC profiles across treatments were statistically determined by ANOSIM (*R* = 0.4134, *P* ≤ 0.037). The stress value representing the accuracy in spatial similarity/dissimilarity was 0.1046. The results of this assay delineated the possible bacterial volatile compounds elicited by QS activity that could be interpreted by mosquitoes, thus triggering specific behaviors.

**Table 2:** *Staphylococcus epidermidis* compounds inhibited by QSI furanone C-30.

**#**	**Compounds**	**Retention time (min)**	Relative abundance^*^	**Quantity range (%)**	**Class**	**References**
			**(Mean** ±**SEM)**			
			**SE** ^a^			
1	4,7-Methano1Hindene^b^	11.86	0.0005 ± 0.0003	87–95		Antibiotic tolerance (Muller et al., 2015)
2	Pregnane-3^c^	37.89	0.8623 ± 0.8194	91		N/A
3	Morphine^b^	40.65	0.7712 ± 0.7007	90–95		QS (Virulence) (Babrowski et al., 2012)
4	Heneicosane	45.64	0.6563 ± 1.1209	95	Alkanes	Attraction for oviposition (Mendki et al., 2000; Navarro-Silva et al., 2009)
5	Heptadecane 9-octyl	48.25	1.5075± 1.4871	89–96		Human breath (Phillips et al., 1999)
6	Tetracosane	49.79	1.0052 ± 1.6381	97	Alkanes	Attraction for oviposition (Torres-Estrada et al., 2007),
7	Lanosta^d^	49.85	0.6936 ± 1.1076	97		N/A

^*^The most abundant Octane as an internal standard is assigned 1 and the others assigned a fractional percent of that value.

^a^SE, *Staphylococcus epidermidis* 1457.

^b^Significant indicator compounds (*P* ≤ 0.05).

^c^Pregnane-3: pregnane-3 20-dione.

^d^Lanosta: 20. Xi.-Lanosta-7, 9(11)-diene-3.beta., 18, 20-triol.

Compounds based on relative frequency and abundance only emitted from *S. epidermidis* and inhibited by QSI treatment from triplicate trials of quadruplicate replicates (*n* = 12) at 25.0 ± 0.5°C with 65.0 ± 5.0% RH.

N/A, not applicable.

### 3.3. Transcriptome alterations or Microbial transcriptome alterations

**Figure 4:**
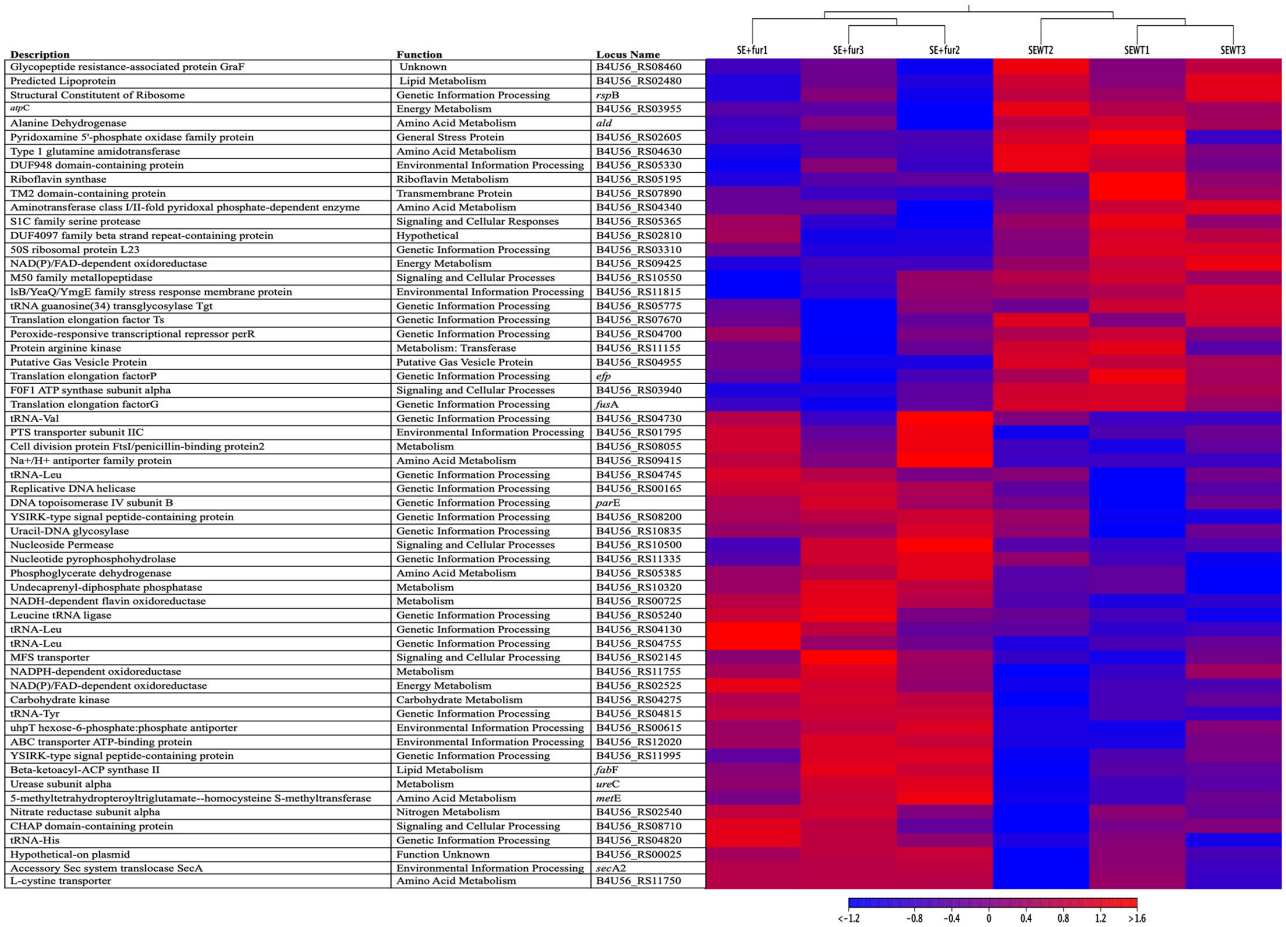
A heat map of gene expression. A heat map of the top 59 significantly expressed genes from triplicate samples (*n* = 3) of *S. epidermidis* 1457 with QSI (SE+fur1-3) and without (SEWT1-3) QSI treatments for 15 min at 25 ± 0.5°C with 65 ± 5.0% RH. The reference genomes were downloaded from the NCBI database using accession numbers CP020462 and CP020463, corresponding to the *S. epidermidis* genome and plasmid, respectively. All genes listed in the heatmap are labeled as listed in RefSeq for a particular gene or locus tag given in the reference genomes' annotation, along with gene descriptions and predicted or known functions.

The messenger RNA molecules expressed by bacterial genes ultimately lead to the production of proteins that influence cellular processes such as volatile production. By analyzing the bacterial transcriptome of fully functional *S. epidermidis* and *S. epidermidis* treated with QSI, we aimed to elucidate which cellular processes are directly linked to bacterial QS functionality. Triplicate samples of *Staphylococcus epidermidis* exposed to furanone (QSI), SE + fur1, SE + fur2, and SE + fur3, and diluent controls not exposed to furanone (*S. epidermidis* wildtype alone, SEwt1, SEwt2, and SEwt3, were processed to elucidate their mRNA expression. Sequencing and trimming yielded an average fragment and read length of 132 and 1,249,203, respectively. Altogether, 65 genes were differentially regulated between *S. epidermidis*+ QSI and *S. epidermidis*. Complete gene lists are shown in Supplementary Table 1. A total of 29 genes were significantly upregulated when *S. epidermidis* was treated with a QSI. A heat map of the top 59 significantly expressed genes from *S. epidermidis* with and without QSI is shown in Figure 4. Of the significantly upregulated genes, four were involved in environmental information processing and included gene encoding for bacterial secretion, lipoprotein export, a hexose-6-phosphate phosphate antiporter, and membrane transport. Seven were involved in genetic information processing, including translation, chaperones, folding catalysts, replication, repair, and (d)NTP-pool sanitation involving *gyrB, dnaB, ung*, and a gene encoding a putative YabN. Twelve were found to be involved in metabolism, including riboflavin metabolism, carbohydrate metabolism, nitrogen or urea metabolism, glycan biosynthesis and metabolism, and lipid metabolism. Moreover, four of these 12 were genes encoding for amino acid transport and metabolism. In addition, three significantly upregulated genes were classified as participating in signaling and cellular processes, including genes encoding a multidrug efflux transporter, a nucleoside transporter, and a gene involved in cell wall metabolism. Finally, one gene from the *S. epidermidis* plasmid p1457, whose function is currently unknown, was upregulated.

A total of 36 genes were significantly downregulated when *S. epidermidis* was treated with a QSI. Five genes were associated with environmental information processing pathways, all of which are involved in response to various stresses, such as general stress response or alkaline shock. In addition, 10 were involved in genetic information processing, with one involved in translation, one in the biosynthesis of the modified nucleoside queuosine tRNA, and three in a stress response. Involvement in stress response included *per*, a gene peroxide-responsive transcriptional repressor; *spxA*, a transcription factor that may function to reduce growth and development processes during periods of stress; and *clpB*, involved in cell recovery from heat, oxidative, and other stress. Moreover, *dps*, a stationary phase nucleoid protein that sequesters iron and protects DNA from damage, was downregulated.

Then, five significantly downregulated genes were associated with metabolism, including three for energy metabolism, one for riboflavin metabolism, and one for encoding a protein arginine kinase associated with general metabolism. In addition, six significantly downregulated genes were associated with signaling and cellular processes, including one encoding a serine protease, one encoding a zinc metallopeptidase, one encoding an M50 family peptidase, and one encoding a RidA family reactive intermediate/imine deaminase. Genes also downregulated and associated with signaling and cellular processes included F0F1 ATP synthase subunit alpha, believed to be involved in cell motility, intracellular trafficking, secretion, vesicular transport, and spoVG, involved in the regulation of cell wall metabolism that plays a role in sporulation and other functions in other organisms. Moreover, we identified two genes encoding two hypothetical proteins and three downregulated genes that were not associated with any of the above pathways, including a gene encoding a predicted lipoprotein, one encoding a putative gas vesicle protein, and one encoding a transmembrane protein. The results of this assay delineated the cellular processes within the bacteria that control the volatile compound production altered by QSI activity and that ultimately led to changes in mosquito behavior.

## 4. Discussion

Bacteria interact with each other and their surroundings through a number of methods, including chemical communication (i.e., QS), by which bacteria respond to the population density of conspecifics and heterospecifics (He et al., 2012). In some instances, other prokaryotic and eukaryotic organisms eavesdrop on this information as a means to interpret their environment (He et al., 2012; Ma et al., 2012). Briefly, it is highly probable that certain densities of bacteria, which are key to initiating QS responses, are monitored by other organisms that utilize that information for their purposes [*sensu lato*, public information (Valone, 1993)]. We determined that *Ae. aegypti* attraction to blood feeders treated with *S. epidermidis* exposed to a QSI resulted in a 55.1% lower attraction to the blood feeders alone when compared with *S. epidermidis* without QSI treatment. Furthermore, the application of QSI reduced *S. epidermidis* VOC profiles by 31.6%, of which some compounds were associated with QS in bacteria, such as morphine (Babrowski et al., 2012; Zhan and French, 2019) and tetracosane (LewisOscar et al., 2018). Transcriptome analysis indicated that treatment with the QSI shifted responses in *S. epidermidis* to increase stress responses, as well as interfere with metabolism and protein synthesis. Our previous study demonstrated differential mosquito behavior associated with the QS mutant, *S. epidermidis arg-*; the *agr* gene expresses an accessory gene regulator for quorum sensing; therefore, removing this gene inhibits quorum sensing of the bacteria (Zhang et al., 2015). Thus, these QS molecules functioned as a cue, possibly a signal, for mosquitoes to locate hosts. This current study, in combination with our previous study (Zhang et al., 2015), provides definitive evidence that QS molecules play an important role in mediating interactions between bacteria and eukaryotes.

Because of the close ties between microbes and their hosts, the ability of bacteria to communicate and behave for social interactions as a multi-cellular organism significantly impacts other organisms (Tomberlin et al., 2012; Zhang et al., 2015). The VOCs produced by host bacteria could be an indicator of a host's nutritional value or other ecologically relevant information. Smeekens et al. (2014) determined that, during disease, specific immune responses (e.g., cytokines) in patients decreased or shifted predominant dermal bacterial populations (Firmicutes: *S. epidermidis*). Therefore, shifted VOC compositions (or concentrations) or the loss of a signature compound resulting from impacted host conditions may induce mosquito host preferences, as such an information could increase the likelihood of securing a blood meal or reduce the likelihood of being killed by the host. The study presented here demonstrated that disrupting a QS circuit within bacteria associated with specific hosts affected gene expression and VOC production and ultimately suppressed mosquito attraction behavior. Organisms ranging from plants (rhizosphere and phyllosphere) to human beings (skin surface and gut) are evolutionarily associated with microbes. Thus, modulation of physical properties within the bacterial environment, such as QS-induced bacterial VOCs (Chernin et al., 2011), opens a new realm of possibilities with regard to the management of medical and veterinary vectors and agricultural pests. Imagine disrupting pathogen spread, neither by the attempted extermination of the vector nor by the elimination of pathogenic microorganisms by resistance-prone methods, but by interrupting the attraction of insect vectors to their host. Such a mechanism could occur by directly modulating the QS pathways of the host's bacteria that trigger their interaction or by isolating the bacterial volatile repertoire activated by the QS pathway for use as a new class of insect attractants (e.g., odor-masking compounds) and repellents (e.g., inhibitory compounds), thereby inhibiting initial pathogen interaction leading to transmission and distribution of the pathogens.

However, additional research is needed to provide more clarity to our conclusion. When examining these mosquito interactions, our approach was to use a single bacterial species and determine its impact on the mosquito behavior of *Ae. aegypti*. Such an approach is limiting in terms of deciphering the true ecological relevance of bacterial interactions with mosquitoes since the bacterial activity in isolation can be quite different than in the community mixtures typically encountered in a complex and dynamic ecosystem (e.g., human skin). Replication of the results in other mosquito species, as well as examining anti-QS activity in *S. epidermidis* by different QSIs (e.g., Syph-1, ethanolic extract) and examining other microbes or communities of microbes with regards to QS engagement, would provide greater insight into the true nature of these interactions influencing mosquito attraction. Furthermore, secondary experiments are needed to determine whether altering the QS responses of host microbes affects the acceptance and blood-feeding behavior of the mosquito. Regardless, this study opens a new door for exploring vector-foraging behavior for hosts and potential mechanisms for reducing pathogen transmission.

## Data availability statement

Original datasets are available in a publicly accessible repository: The original contributions presented in the study are publicly available. This data can be found here: https://www.ncbi.nlm.nih.gov/bioproject/PRJNA907831.

## Author contributions

TC and JT: conceptualization. DK, HJ, TC, and JT: methodology, analyses, and review and editing. DK and TC: writing—original draft. All authors contributed to the article and approved the submitted version.
