# Supplementary material for: Quorum sensing gene regulation in Staphylococcus epidermidis reduces the attraction of Aedes aegypti (L.) (Diptera: Culicidae)
Source: Front Microbiol. 2023 Jun 22;14:1208241. doi: 10.3389/fmicb.2023.1208241 (PMC10324375; doi:10.3389/fmicb.2023.1208241)
Supplement: Supplementary file 1 [file Data_Sheet_1.pdf]

Supplementary Material

• Host Bacterial Quorum Sensing Signaling Regulates Mosquito-Feeding Behavior

Dongmin Kim[1]‡, Tawni L. Crippen[2*], Heather R. Jordan[3], Jeffery K. Tomberlin

*** Correspondence:**
Tawni L. Crippen, PhD
tc.crippen@usda.gov

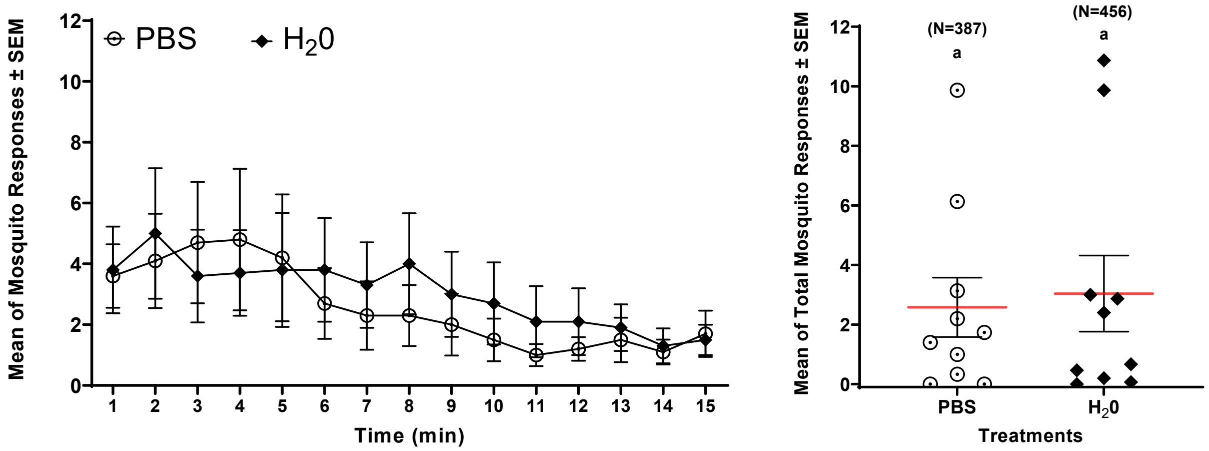

Supplementary Figure 1. Dual Choice Assay Responses to PBS and H2O. (Left panel) Mean number of 3-5 d-old (post-emergence) female *Ae. aegypti* mosquito responses per minute ± SEM to H_2O (blood-feeders treated with reverse osmosis water) or PBS (blood-feeders treated with phosphate buffered saline), placed at equal distance horizontally and vertically (24 cm) apart on the top of an 82 cm (L) x 45 cm (W) x 52 cm (H) Plexiglas cage during the experiments of 15 min with 50 mosquitos at 24 °C and 65% RH. (Right panel) Mean number of mosquito responses for a 15 min assay period. Each dot represents an individual mean for a single replication (n=10). The solid red lines and vertical lines indicated a group mean and standard error, respectively. The same letter is not significantly different ($P \leq 0.05$).

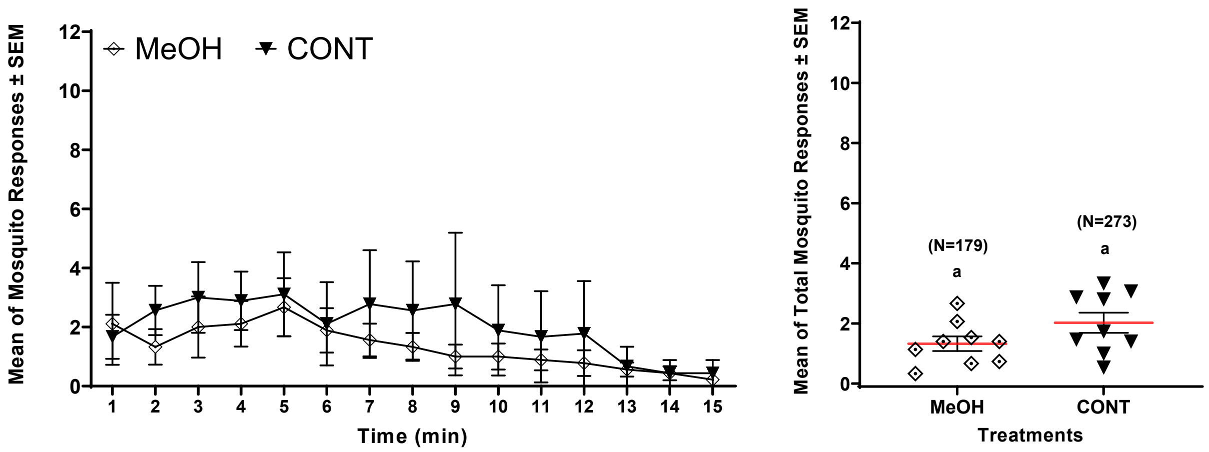

Supplementary Figure 2. Dual Choice Assay Responses to Methanol. (Left panel) Mean number of 3-5 d-old (post-emergence) female *Ae. aegypti* mosquito responses per minute ± SEM to MeOH (blood-feeders treated with Methanol) or with CONT (blood feeder), placed at equal distance horizontally and vertically (24 cm) apart on the top of an 82 cm (L) x 45 cm (W) x 52 cm (H) Plexiglas cage during the experiments of 15 min with 50 mosquitos at 24 °C and 65% RH. (Right panel) Mean number of mosquito responses for a 15 min assay period. Each dot represents an individual mean for a single replication (n=9). The solid red lines and vertical lines indicated a group mean and standard error, respectively. The same letter is not significantly different ($P \leq 0.05$).

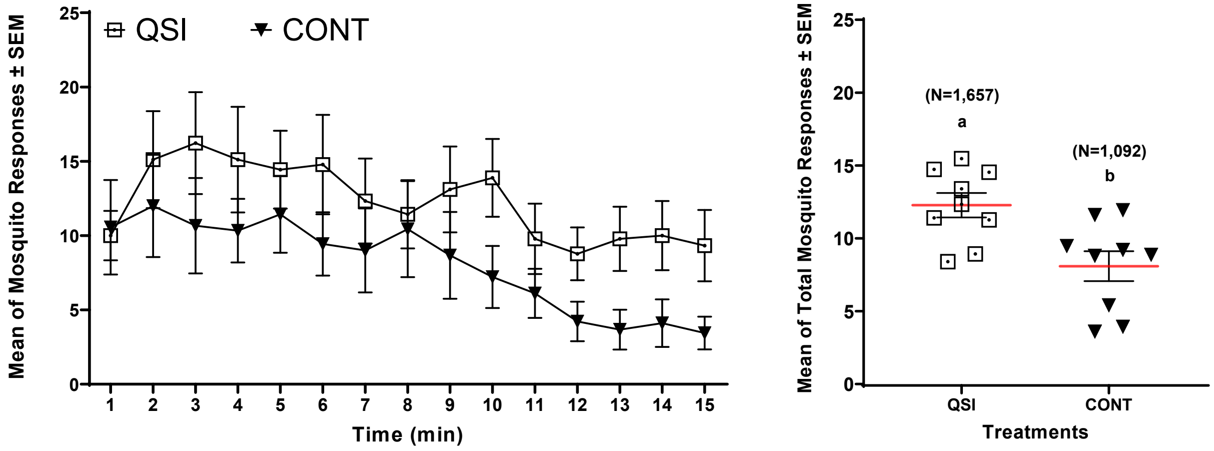

Supplementary Figure 3. Dual Choice Assay Responses to QSI Furanone C-30. (Left panel) Mean number of 3-5 d-old (post-emergence) female *Ae. aegypti* mosquito responses per minute ± SEM to QSI (blood-feeders treated with quorum sensing inhibitor furanone C-30) or with CONT (blood feeder), placed at equal distance horizontally and vertically (24 cm) apart on the top of an 82 cm (L) x 45 cm (W) x 52 cm (H) Plexiglas cage during the experiments of 15 min with 50 mosquitos at 24 °C and 65% RH. (Right panel) Mean number of mosquito responses for a 15 min assay period. Each dot represents an individual mean for a single replication (n=9). The solid red lines and vertical lines indicated a group mean and standard error, respectively. The same letter is not significantly different ($P \leq 0.05$).

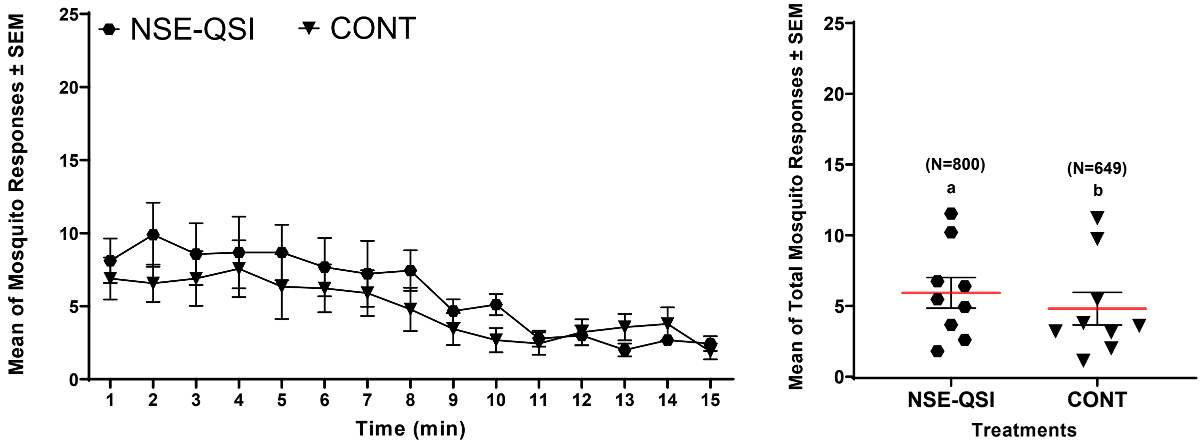

Supplementary Figure S4. Dual Choice Assay Responses to Co-culture of SE and QSI Furanone C-30. (Left panel) Mean number of 3-5 d-old (post-emergence) female *Ae. aegypti* mosquito responses per minute ± SEM to NSE-QSI (blood-feeders treated with co-culture of *S. epidermidis* 1457 and quorum sensing inhibitor not in physical contact) or with CONT (blood feeder), placed at equal distance horizontally and vertically (24 cm) apart on the top of an 82 cm (L) x 45 cm (W) x 52 cm (H) Plexiglas cage during the experiments of 15 min with 50 mosquitos at 24 °C and 65% RH. (Right panel) Mean number of mosquito responses for a 15 min assay period. Each dot represents an individual mean for a single replication (n=9). The solid red lines and vertical lines indicated a group mean and standard error, respectively. The same letter is not significantly different ($P \leq 0.05$).
