# Supplementary material for: Quorum sensing gene regulation in Staphylococcus epidermidis reduces the attraction of Aedes aegypti (L.) (Diptera: Culicidae)
Source: Front Microbiol. 2023 Jun 22;14:1208241. doi: 10.3389/fmicb.2023.1208241 (PMC10324375; doi:10.3389/fmicb.2023.1208241)
Supplement: Supplementary file 2 [file Table_1.pdf]

Supplementary Table 1: Complete list of transcriptome genes differentially regulated between SE+ QSI and SE.

Name	Chromosome	Region	Max group mean	Fold change	P-value	Description	Protein ID	Pathway	Gene Name	Additional Information
Up-Regulated										
B4U56_RS00725	NZ_CP020463.1	130389..133406	9.14	2.69	0.00	NADH-dependent flavin oxidoreductase	WP_002484651.1	metabolism		riboflavin metabolism
B4U56_RS11750	NZ_CP020463.1	2388296..2389684	11.02	2.93	0.01	L-cystine transporter	WP_001829358.1	Amino acid metabolism		Cysteine transport
B4U56_RS04275	NZ_CP020463.1	866485..867444	6.12	4.53	0.04	carbohydrate kinase	WP_002468351.1	Carbohydrate metabolism		
metE	NZ_CP020463.1	2409602..2411848	5.81	3.16	0.03	5-methyltetrahydropteroyltriglutamate homocysteine S-methyltransferase	WP_002437223.1	Amino acid metabolism	metE	Cysteine and methionine biosynthesis
B4U56_RS02525	NZ_CP020463.1	541507..543912	4.86	3.33	0.02	NAD(P)/FAD-dependent oxidoreductase	WP_002438309.1	Energy metablism		oxidative phosphorylation
secA2	NZ_CP020463.1	253194..255584	5.18	3.02	0.02	accessory Sec system translocase SecA2	WP_001832960.1	Environmental Information Processing	SecA2	Membrane transport; Bacterial secretion system
B4U56_RS12020	NZ_CP020463.1	2435981..2436739	6.98	7.04	0.03	ABC transporter ATP-binding protein	WP_001831790.1	Environmental Information Processing	lolD	ABC-type lipoprotein export system, ATPase
B4U56_RS01795	NZ_CP020463.1	384686..385744	5.65	4.53	0.04	PTS transporter subunit IIC	WP_001830678.1	Environmental Information Processing		membrane transport
B4U56_RS04795	NZ_CP020463.1	963315..963407	624.08	1.45	0.03	amino acid ABC transporter permease	WP_002468258.1	Amino acid metabolism	hisM	histidine transport
B4U56_RS00615	NZ_CP020463.1	102226..103605	11.14	2.65	0.01	hexose-6-phosphate:phosphate antiporter	WP_002486119.1	Environmental Information Processing	uhpT	Regulates transport of inorganic phosphate sugars
B4U56_RS00025	NZ_CP020462.1	2041..2790	11.02	2.78	0.04	hypothetical --on plasmid	WP_002470105	Found on plasmid-function unknown		Domain found in lipoproteins
B4U56_RS05240	NZ_CP020463.1	1030914..1033358	6.44	2.51	0.01	leucine--tRNA ligase	WP_002440239.1	Genetic Information Processing		Translation
B4U56_RS08200	NZ_CP020463.1	1692244..1694904	15.07	1.68	0.01	YSIRK-type signal peptide-containing protein	WP_083043965.1	Genetic Information Processing		Chaperones and folding catalysts
B4U56_RS11995	NZ_CP020463.1	complement(2426607..2431172)	5.95	1.99	0.01	YSIRK-type signal peptide-containing protein	WP_083043968.1	Genetic Information Processing		Chaperones and folding catalysts
parE	NZ_CP020463.1	complement(1469624..1471624)	5.57	2.73	0.04	DNA topoisomerase IV subunit B	WP_001831078.1	Genetic Information Processing	gyrB	Replication and repair
B4U56_RS10835	NZ_CP020463.1	complement(2201412..2202062)	13.71	2.97	0.04	uracil-DNA glycosylase	WP_001832132.1	Genetic Information Processing	ung	Replication and Repair-base excision repair
B4U56_RS11335	NZ_CP020463.1	complement(2299795..2300985)	5.99	3.80	0.03	nucleotide pyrophosphohydrolase	WP_001832236.1	Genetic Information Processing	yabN	Chromosome and other proteins
B4U56_RS10320	NZ_CP020463.1	2104470..2105342	8.74	4.12	0.02	undecaprenyl-diphosphate phosphatase	WP_001832082.1	Metabolism		Glycan biosynthesis and metabolism
B4U56_RS05385	NZ_CP020463.1	complement(1077302..1078897)	4.84	4.12	0.02	phosphoglycerate dehydrogenase	WP_001830903.1	Amino acid metabolism		Glycine, serine and threonine metabolism
fabF	NZ_CP020463.1	complement(1885119..1886363)	11.35	2.05	0.04	beta-ketoacyl-[acyl-carrier-protein] synthase II	WP_001829324.1	Lipid Metabolism	fabF	Fatty Acid biosynthesis
B4U56_RS08055	NZ_CP020463.1	complement(1664343..1666670)	6.66	3.70	0.00	PASTA domain-containing protein; Cell division protein FtsI/penicillin-binding protein 2	WP_002439409.1	Metabolism	pbp2	Glycan biosynthesis and metabolism
ureC	NZ_CP020463.1	complement(655898..657613)	10.32	2.06	0.03	urease subunit alpha	WP_001832399.1	metabolism	ureC	purine metabolism/urea metabolism
B4U56_RS02540	NZ_CP020463.1	545334..549017	6.17	2.27	0.02	nitrate reductase subunit alpha	WP_002470190.1	Nitrogen metabolism	narG	Dissimilatory nitrate reduction, nitrate => ammonia
B4U56_RS09415	NZ_CP020463.1	complement(1931021..1932337)	4.57	28.04	0.04	Na+/H+ antiporter family protein"	WP_001831915.1	Amino acid metabolism	YuiF	Putative amino acid transporter
B4U56_RS00165	NZ_CP020463.1	20533..21933	13.83	2.42	0.01	replicative DNA helicase	WP_001831779.1	Genetic Information Processing	dnaB	Replication and repair
B4U56_RS11755	NZ_CP020463.1	complement(2389808..2390572)	10.68	3.42	0.02	NADPH-dependent oxidoreductase	WP_011082826.1	metabolism		riboflavin metabolism
B4U56_RS02145	NZ_CP020463.1	458536..459810	13.29	2.46	0.01	Bcr/CflA family efflux MFS transporter	WP_002438271.1	Signalling and Cellular Components		multidrug efflux transporter
B4U56_RS10500	NZ_CP020463.1	complement(2138251..2139480)	6.30	4.12	0.03	nucleoside permease"	WP_002499043.1	Signalling and Cellular Components	nupC	Transporters
B4U56_RS08710	NZ_CP020463.1	complement(1794046..1795509)	7.94	2.88	0.05	CHAP domain-containing protein	WP_032606340.1	Signalling and Cellular Components	PGRP	Peptidoglycan recognition protein-possible autolysin
Down-Regulated										
glmS	NZ_CP020463.1	755938..757743	41.28	-1.43	0.02	glutamine--fructose-6-phosphate transaminase	WP_001829889.1	Amino Acid metabolism		Alanine, aspartate and glutamate metabolism
B4U56_RS04340	NZ_CP020463.1	complement(883991..885277)	7.44	-4.04	0.01	aminotransferase class I/II-fold pyridoxal phosphate-dependent enzyme	WP_002498971.1	Amino Acid metabolism		Aminotransferase
B4U56_RS04630	NZ_CP020463.1	941044..941562	87.83	-1.86	0.00	type 1 glutamine amidotransferase	WP_001830414.1	Amino Acid metabolism		Aminotransferase
ald	NZ_CP020463.1	1094329..1095444	45.49	-1.55	0.01	alanine dehydrogenase	WP_001830775.1	Amino Acid metabolism		Alanine degradation
gap	NZ_CP020463.1	complement(1999664..2000674)	97.48	-1.48	0.01	type 1 glyceraldehyde-3-phosphate dehydrogenase	WP_001829667.1	Metabolism-transferase		Carbohydrate Metabolism
B4U56_RS03955	NZ_CP020463.1	805810..806214	13.62	-4.21	0.05	Ftype H1 ATP synthase subunit epsilon	WP_001829924.1	Energy Metabolism	atpC	H+-transporting ATP synthase epsilon chain
B4U56_RS09425	NZ_CP020463.1	complement(1934324..1935532)	35.28	-2.51	0.00	AD(P)/FAD-dependent oxidoreductase	WP_001831966.1	Energy Metabolism		
B4U56_RS05530	NZ_CP020463.1	1112273..1113394	73.77	-1.42	0.04	Citrate Synthase	WP_001830889.1	Energy Metabolism		
B4U56_RS11815	NZ_CP020463.1	complement(2397591..2397845)	51.02	-2.79	0.02	GlsB/YeaQ/YmgE family stress response membrane protein	WP_001832461.1	Environmental Infoamtion Processing		general stress response
B4U56_RS02605	NZ_CP020463.1	complement(560057..560521)	59.85	-1.71	0.03	General Stress Protein	WP_011082779.1	Environmental Infoamtion Processing		general stres response
B4U56_RS03535	NZ_CP020463.1	725048..725629	112.59	-1.42	0.03	alkaline shock response membrane anchor protein, amaP	WP_002438599.1	Environmental Infoamtion Processing	amaP	alkaline stress response; anchors Asp23
B4U56_RS05330	NZ_CP020463.1	1062803..1063294	41.09	-2.33	0.02	DUF948 domain-containing protein	WP_001830868.1	Environmental Infoamtion Processing	yoxC	general stres response
B4U56_RS03310	NZ_CP020463.1	694009..694284	17.21	-6.60	0.04	50S ribosomal protein L23	WP_001829755.1	Genetic Information Prcessing		Translation
B4U56_RS05775	NZ_CP020463.1	1164496..1165635	8.83	-3.46	0.01	tRNA guanosine(34) transglycosylase Tgt	WP_001830840.1	Genetic Information Prcessing	tgt	modified nucleotide tRNA biogenesis
B4U56_RS04700	NZ_CP020463.1	955901..956353	31.67	-2.25	0.04	peroxide-responsive transcriptional repressor, PerR	WP_001830391.1	Genetic Information Prcessing	PerR	Transcription Regulator
efp	NZ_CP020463.1	1269201..1269758	24.89	-2.04	0.04	elongation factor P	WP_002485096.1	genetic Information Prcessing	efp	Translation
B4U56_RS07670	NZ_CP020463.1	complement(1578795..1579673)	39.05	-1.82	0.01	elongation factor Ts	WP_032606451.1	Genetic Information Prcessing		Translation
fusA	NZ_CP020463.1	complement(2239817..2241898)	28.32	-1.60	0.00	elongation factor G	WP_001832287.1	Genetic Information Prcessing	fusA	Translation
rpsB	NZ_CP020463.1	complement(1579825..1580613)	60.22	-1.54	0.02	30S ribosomal protein S2	WP_001832557.1	Genetic Information Prcessing		Translation
spxA	NZ_CP020463.1	complement(1877012..1877407)	375.09	-1.41	0.03	transcriptional regulator Spx	WP_001829294.1	Genetic Information Prcessing	spxA	Global transcripton control during oxidative stress
B4U56_RS03760	NZ_CP020463.1	768008..768454	376.46	-1.36	0.04	DNA starvation/stationary phase protection protein	WP_001829900.1	Genetic Information Prcessing	dps	stationary phase nucleoid protein that sequesters iron and protects DNA from damage
clpB	NZ_CP020463.1	complement(1889532..1892141)	161.22	-1.36	0.03	ATP-dependent chaperone ClpB	WP_002467691.1	Genetic Information Prcessing		Part of a stress induced multichaperone system
B4U56_RS11375	NZ_CP020463.1	complement(2311115..2311495)	137.97	-1.41	0.05	RidA family protein	WP_032606419.1	Signaling and Cellular processes		
B4U56_RS05365	NZ_CP020463.1	complement(1072179..1073417)	21.08	-1.67	0.04	serine protease	WP_001830793.1	signaling and Cellular processes		
B4U56_RS06640	NZ_CP020463.1	1331037..1331732	260.03	-1.44	0.01	zinc metallopeptidase	WP_001831045.1	signaling and Cellular processes		
B4U56_RS11155	NZ_CP020463.1	complement(2267909..2268916)	40.92	-2.04	0.00	Protein Arginine Kinase	WP_002438663.1	metabolism-transferase		
B4U56_RS02480	NZ_CP020463.1	534117..534485	13.71	-6.61	0.04	DUF4467 domain-containing protein	WP_011082785.1	predicted lipoprotein		
B4U56_RS04955	NZ_CP020463.1	complement(988172..988543)	26.40	-3.29	0.02	YtxH domain-containing protein	WP_002456353.1	Putative Gas Vesicle Protein		one study showing association with quorum sensing
B4U56_RS05195	NZ_CP020463.1	1022271..1022903	21.45	-2.22	0.04	riboflavin synthase	WP_001830723.1	riboflavin metabolism		
B4U56_RS10550	NZ_CP020463.1	2148437..2149183	25.82	-2.43	0.01	M50 family peptidase	WP_002438814.1	Signaling and Cellular processes		
B4U56_RS03940	NZ_CP020463.1	801894..803405	12.88	-1.80	0.05	F0F1 ATP synthase subunit alpha	WP_001829913.1	Signaling and Cellular processes		Cell motility, Intracellular trafficking, secretion, and vesicular transport
B4U56_RS05460	NZ_CP020463.1	complement(1093681..1094181)	162.32	-1.50	0.01	Universal Stress Protein A	WP_002494483.1	Environmental Infoamtion Processing	uspA	
B4U56_RS11370	NZ_CP020463.1	complement(2310747..2311055)	405.06	-1.49	0.01	septation protein SpoVG	WP_001832195.1	Signaling and Cellular processes	SpoVG	sporulation in Bacillus; protein transport and capsular formation
B4U56_RS07890	NZ_CP020463.1	1628029..1628232	123.87	-1.83	0.02	TM2 domain-containing protein	WP_001830141.1	transmembrane protein		
B4U56_RS08460	NZ_CP020463.1	1745803..1745937	70.53	-2.62	0.04	hypothetical				
B4U56_RS02810	NZ_CP020463.1	602122..602925	19.14	-2.28	0.02	hypothetical				
